# Global Reconstruction of Naturalized River Flows at 2.94 Million Reaches

**DOI:** 10.1029/2019WR025287

**Published:** 2019-08-05

**Authors:** Peirong Lin, Ming Pan, Hylke E. Beck, Yuan Yang, Dai Yamazaki, Renato Frasson, Cédric H. David, Michael Durand, Tamlin M. Pavelsky, George H. Allen, Colin J. Gleason, Eric F. Wood

**Affiliations:** ^1^ Department of Civil and Environmental Engineering Princeton University Princeton NJ USA; ^2^ State Key Laboratory of Hydroscience and Engineering, Department of Hydraulic Engineering Tsinghua University Beijing China; ^3^ Institute of Industrial Science The University of Tokyo Tokyo Japan; ^4^ School of Earth Sciences The Ohio State University Columbus OH USA; ^5^ Jet Propulsion Laboratory California Institute of Technology Pasadena CA USA; ^6^ Department of Geological Sciences University of North Carolina at Chapel Hill Chapel Hill NC USA; ^7^ Department of Geography Texas A&M University College Station TX USA; ^8^ Department of Civil and Environmental Engineering University of Massachusetts Amherst Amherst MA USA

## Abstract

Spatiotemporally continuous global river discharge estimates across the full spectrum of stream orders are vital to a range of hydrologic applications, yet they remain poorly constrained. Here we present a carefully designed modeling effort (Variable Infiltration Capacity land surface model and Routing Application for Parallel computatIon of Discharge river routing model) to estimate global river discharge at very high resolutions. The precipitation forcing is from a recently published 0.1° global product that optimally merged gauge‐, reanalysis‐, and satellite‐based data. To constrain runoff simulations, we use a set of machine learning‐derived, global runoff characteristics maps (i.e., runoff at various exceedance probability percentiles) for grid‐by‐grid model calibration and bias correction. To support spaceborne discharge studies, the river flowlines are defined at their true geometry and location as much as possible—approximately 2.94 million vector flowlines (median length 6.8 km) and unit catchments are derived from a high‐accuracy global digital elevation model at 3‐arcsec resolution (~90 m), which serves as the underlying hydrography for river routing. Our 35‐year daily and monthly model simulations are evaluated against over 14,000 gauges globally. Among them, 35% (64%) have a percentage bias within ±20% (±50%), and 29% (62%) have a monthly Kling‐Gupta Efficiency ≥0.6 (0.2), showing data robustness at the scale the model is assessed. This reconstructed discharge record can be used as a priori information for the Surface Water and Ocean Topography satellite mission's discharge product, thus named “Global Reach‐level A priori Discharge Estimates for Surface Water and Ocean Topography”. It can also be used in other hydrologic applications requiring spatially explicit estimates of global river flows.

## Key Points

- Thirty‐five‐year global reconstruction of river flows at unprecedented 2.94 million reaches extracted from 90‐m high‐accuracy DEM
- Novel bias correction (“sparse CDF matching”) is conducted grid‐by‐grid against machine learning‐derived global runoff characteristics maps
- Evaluation against >14,000 gauges shows data robustness for hydrologic applications and utility in spaceborne discharge estimation efforts

## 1 Introduction

Reconstructing historical river flows across the full spectrum of stream orders is of vital importance for scientific understanding of global water and biogeochemical cycles (Downing, 2012; Oki & Kanae, 2006) and the greenhouse gas contribution from rivers to the global carbon budget (Catalán et al., 2016; Lauerwald et al., 2015; Marx et al., 2017; Raymond et al., 2013). It is also of tremendous practical value to be able to predict global river flows at hyperresolution with appropriate lead times (Bierkens et al., 2015; Wood et al., 2011) to inform early flood and drought warnings. However, adequate reconstruction and prediction of global river flow dynamics is still hindered by many challenges in front of the research communities.

Stream gauging is the most accurate way to measure river discharge, but the spatial coverage of global gauge observations is largely limited for practical, economic, and political reasons (Fekete & Vörösmarty, 2007). Recent decreases in active stream gauges (Hannah et al., 2011) have exacerbated the problem especially for understanding the hydrology in ungauged basins (Sivapalan, 2003). Hydrologic modeling can be used to fill in the spatial gaps and predict flows in ungauged basins. However, the performance of hydrologic modeling often suffers from the lack of high‐quality global data sets for modeling inputs (Beck et al., 2019), optimal combination of physical parameterization schemes (Zheng et al., 2018), reliable approaches to estimating model parameters (Samaniego et al., 2010), and computational power to represent physical realism at sufficiently high resolutions (Bierkens et al., 2015). In recent decades, global river modeling has seen advancements in better physical representations of floodplain and hillslope processes (Li et al., 2013; Yamazaki et al., 2011), improved routing methods (Bates et al., 2010; Häfliger et al., 2015; Ngo‐Duc et al., 2007), incorporation of human activity and water management modules (Hanasaki et al., 2006; Sutanudjaja et al., 2018; Zajac et al., 2017), and improved precipitation forcing data from satellites (Wu et al., 2014). However, often research emphasizes a single aspect of the hydrologic modeling chain, that is, forcing, land surface models (LSMs), or routing, while attempts to systematically reduce the uncertainty from all components have been relatively rare. In addition, although the coarse channel representations used by pioneering global routing studies (Lohmann et al., 1998; Miller et al., 1994; Oki & Sud, 1998) have been improved in recent studies, these have not yet advanced to a scale that pertains to “locally relevant” societal needs (Bierkens et al., 2015) and a scale matching satellite depiction of the global water surfaces (Allen & Pavelsky, 2018; Pekel et al., 2016). For example, the finest channel resolution in previous global river modeling studies is at the level of kilometers, for example, 5 arcmin (~8 km; Sutanudjaja et al., 2018), 1/16° (~6 km; Li et al., 2013), and 15 arcmin (~25 km; Yamazaki et al., 2011). However, rivers are highly sinuous geomorphic features and require much finer depictions at a resolution of meters to tens of meters to preserve the true planform flow geometry, and this scale has not been pinned down by existing global river routing models. Several studies have used vector‐based river network for realistic channel representations while being computationally efficient (David et al., 2011; Lehner & Grill, 2013; Yamazaki et al., 2013). These vector‐based models are very promising, but they have yet to be implemented at the global scale.

Recent progress in remote sensing (RS) offers promising alternatives to monitoring global river discharge from space (Bjerklie et al., 2018; Dijk et al., 2016; Durand et al., 2016; Gleason et al., 2014; Gleason & Smith, 2014; Hagemann et al., 2017; Tarpanelli et al., 2013). These RS progresses may particularly benefit regions with complex processes where simple extrapolations of model physical parameterizations may raise problems. Although collected less frequently than desired for daily streamflow estimation, RS can also greatly complement the stream gauge observations in terms of spatial coverage. However, the conversion of RS observations to streamflow estimates is also subject to large uncertainties (e.g., Biancamaria et al., 2010; Durand et al., 2016; Gleason et al., 2014). For example, the upcoming Surface Water and Ocean Topography (SWOT) mission, scheduled for launch in 2021, will provide global estimates of river discharge derived exclusively from its interferometer measurements, which will set a new standard in RS (Biancamaria et al., 2016). Based on SWOT's expected observations of river width, height, and slope, a range of discharge algorithms have been developed and intercompared to evaluate the strengths and weaknesses of each algorithm in different climatic and hydraulic settings (Durand et al., 2016). Note that as SWOT is not yet launched, SWOT discharge algorithms were developed and tested using either hydraulic model outputs (e.g., Bonnema et al., 2016; Durand et al., 2016), estimates of river width and/or height from current satellites (Bjerklie et al., 2018; Gleason et al., 2014; Hagemann et al., 2017), or AirSWOT, an airborne variant of the SWOT instrument (e.g., Tuozzolo et al., 2019). However, due to the underconstrained nature of the estimation problem (i.e., solving for discharge without knowing channel geometry and friction parameter), existing SWOT discharge algorithms improve as prior knowledge of river discharge improves (Durand et al., 2016). For example, Tuozzolo et al. (2019) recently tested three algorithms on AirSWOT data collected over the Willamette River in Oregon, USA, and found that all three algorithms were sensitive to the initial estimates of mean annual discharge used to begin the estimation process. While the importance of initial estimates of river flows is highlighted, at the global scale, models remain the only tool for providing such estimates, and, at present, hydrologic modeling outputs from the Water Balance Model (WBM; Wisser et al., 2010) or WBMsed (Cohen et al., 2014) are used to provide prior information to current SWOT algorithms. However, the model is run at relatively low resolution (5‐arcmin at global scale), and there is a lack of spatially explicit understanding of the skill of the WBM‐simulated discharge. This limitation potentially hampers future SWOT discharge accuracy for global rivers, and it has become increasingly clear that adequate modeling must be separately conducted so that the novel RS measurements such as those from SWOT can improve the best‐possible current understanding of global flows.

To provide an improved a priori estimate of global river flows in support of the SWOT mission and other scientific applications requiring spatiotemporally continuous discharge estimates, we aim to develop an advanced modeling framework that simulates reach‐scale river discharge based on realistically represented channel networks. The modeling framework attempts to address the challenges facing the field of global river modeling (i.e., coarse channel representations and a lack of systematic uncertainty reduction) while considering the specific needs of SWOT. A range of state‐of‐the‐art global data sets and novel modeling practices are developed and synthesized to achieve the optimal model performance (section 2). The 35‐year reconstruction of daily and monthly streamflow records (1979 to 2014) are comprehensively assessed using observations from over 14,000 stream gauges globally, covering the full spectrum of stream orders, and the aim is to inform, to the maximum extent possible, the capabilities and limitations of the modeling database. Lastly, we present the final modeling results in the context of SWOT‐observable river reaches as an example to illustrate the potential applications of the updated global flow record.

The paper is organized as follows: The models, data, and newly developed methods of this study are described in section 2. Section 3 presents the modeling results with comprehensive global assessments, analyses of methods to improve the model simulation, analyses of factors potentially influencing the model performance, followed by discussing the utility of the results to the SWOT mission. Section 4 closes with major conclusions and suggestions for future work.

## 2 Data, Models, and Methods

### 2.1 Hydrologic Modeling Workflow

**Figure 1:**
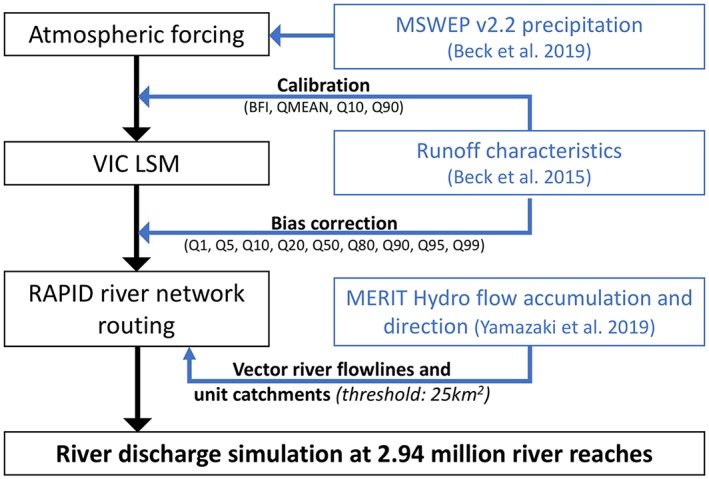
The hydrologic modeling workflow and data sets to constrain the global simulation. VIC = Variable Infiltration Capacity; LSM = land surface model; RAPID = Routing Application for Parallel computatIon of Discharge; MSWEP = Multi‐Source Weighted‐Ensemble Precipitation.

Streamflow modeling sits at the end of the hydrologic modeling chain consisting of atmospheric forcing, LSMs, and a river routing model (Figure 1, black boxes). Therefore, its modeling uncertainty can stem from all these upstream components. To constrain the uncertainty as much as possible, we use the state‐of‐the‐art datasets available at the global scale and a range of newly developed modeling practices as detailed below (blue boxes in Figure 1):

1. For the precipitation forcing, a recently published 0.1° and 3‐hourly precipitation data set that optimally merges a range of gauge‐, reanalysis‐, and satellite‐based precipitation with full global coverage (Beck et al., 2019) is used. Other forcing variables (including min/max 2‐m air temperatures and 10‐m wind speed) are obtained from the Climate Forecasting System Reanalysis.
2. For runoff simulation, the Variable Infiltration Capacity (VIC) LSM (Liang et al., 1994) is used, where model parameter calibration and bias correction (BC) are performed against machine learning (ML)‐derived, global runoff characteristic maps (Beck et al., 2015; hereafter *Q*_c_ maps). More specifically, Beck et al. (2015) regionalized runoff signatures (i.e., runoff values at specific levels) to the global scale by regressing discharge observations from 3,000–4,000 naturalized catchments to 20 climatic and physiographic predictors through neural network training, which generated 17 global *Q*_c_ maps. Motivated by these maps constraining runoff signatures in ungauged basins, new calibration approaches have been developed and tested for the VIC LSM. In this study, instead of directly calibrating VIC against limited gauge observation as in a traditional approach, we calibrate VIC at each 0.25° grid cell (a total of ~0.24 million grid cells for the globe) independently, using the baseflow index, climatology runoff (*Q*_MEAN_), and runoff percentiles (*Q*_10_ and *Q*_90_) as the reference. Three sensitive VIC parameters controlling the generation of surface and subsurface flow are selected for calibration (see Text S1 in the supporting information), including the variable infiltration curve parameter (*bi*), thickness of soil layer 2 (*thick2*), and fraction of the maximum velocity of base flow at which nonlinear base flow begins (*Ds*). The Shuffled Complex Evolution (SCE‐UA) algorithm is employed to find the optimal parameter set for each grid cell. The objective function for each grid cell was as follows:
  $$\mathrm{Obj}=w_{1}\left|\ln\left(\frac{Q{\text{MEAN}}_{m}}{Q{\text{MEAN}}_{o}}\right)\right|+w_{2}\left|\ln\left(\frac{{\mathrm{BFI}}_{m}}{{\mathrm{BFI}}_{o}}\right)\right|+w_{3}\left|\ln\left(\frac{Q_{10m}}{Q_{10o}}\right)\right|+w_{4}\left|\ln\left(\frac{Q_{90m}}{Q_{90o}}\right)\right|$$
  where subscript *o* represents GSCD values and subscript *m* represents values calculated from the model simulation. *w*_i_(*i* = 1,2,3,4) represents the weighting factor to indicate the importance of each runoff characteristics during calibration, and it is set to {0.6, 0.2, 0.1, 0.1} for the four characteristics in this study. More details of this calibration method will be illustrated in a companion paper. The spatially distributed global VIC parameters after calibration are shown in Figures S1–S3. Here we develop a new postprocessing approach to further reduce the model biases that are still present after calibration, and details of the new BC method named “sparse cumulative density function (CDF) matching” are provided in section 2.2.
3. For the river network routing, the Routing Application for Parallel computatIon of Discharge (RAPID; David et al., 2011; David, 2019) is used due to its flexibility in dealing with vector river networks in a range of regional‐ to continental‐scale applications (David et al., 2013; Lin, Hopper, et al., 2018; Lin, Rajib, et al., 2018; Tavakoly et al., 2017). Global extraction of the vector river flowlines used for RAPID routing (~2.94 million, covering 60°S to 90°N) are described in section 2.3. Below, the RAPID configuration and model parameters and its spatial mapping with the VIC LSM are briefly summarized.

The RAPID model solves a matrix form of the Muskingum method, which requires two parameters (*k* and *x*) to be estimated for each vector river flowline: while *x* is a dimensionless weighting factor less sensitive in Muskingum and is set to 0.3 globally, *k* is a flood wave travel time parameter that can play a key role in the routing performance. To reasonably estimate *k* for the 2.94 million river reaches used for routing, the following steps are undertaken. Those include (1) running RAPID with *Q*_MEAN_ and arbitrarily set *k* and *x* to generate *Q*_clim_ for all river reaches, (2) using *Q*_clim_ to estimate river width (*w*) and depth (*d*) following a long‐established power law equation recently used by Andreadis et al. (2013) to general global data set, (3) using Manning's equation to estimate flow celerity (*c*) with *w*, *d*, channel slope, and Manning's roughness assumed 0.035, similar to Allen, David, et al. (2018), and (4) using channel length (*L*) and *c* to calculate flood wave travel time (*L/c*) to obtain *k* for each river reach. To map the VIC gridded runoff (0.25°) onto the vectorized hydrography (section 2.3) to determine the lateral inflows to RAPID, an area‐weighted mapping technique (Lin, Yang, et al., 2018) is adopted, which is demonstrated to perform better than a simple centroid‐based runoff extraction technique used in earlier studies (Lin, Rajib, et al., 2018). The RAPID model also requires the global river network topology (i.e., connectivity) to be specified as inputs. These processes are all established with Python scripting for computationally efficient global implementation, which is flexible to deal with any grid‐based LSM and vector hydrography (see shared Python scripts at https://github.com/peironglinlin/GlobalHydro/). The spin‐up time for VIC and RAPID are both five years, and the internal time step are both 3‐hourly; the modeled discharge is averaged to daily and monthly values for final model evaluation (sections 3.1 to 3.3).

### 2.2 A New Approach for BC: Sparse CDF Matching

To deal with the model biases that may still be present after LSM calibration (e.g., biases related to errors from precipitation forcing, LSM structures/parameterizations, and underrepresented routing processes), we propose a new BC approach that corrects the VIC runoff biases referenced against nine *Q*_c_ maps also delivered by Beck et al. (2015). The problem is very similar to the CDF matching used in a traditional BC (Reichle & Koster, 2004), except here no full CDF is available except for some sparse percentile values (*Q*_c_). Our assumption is that these *Q*_c_ maps trained from ML can potentially offer useful information on runoff signatures beyond our limited knowledge of model processes and parameters, which is in line with the increasing recognition that considers ML as a powerful approach to understand hydrology in ungauged basins (e.g., Zhang et al., 2018).

**Figure 2:**
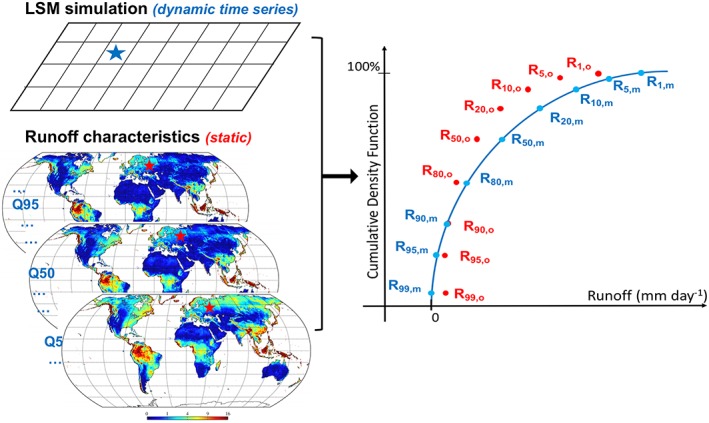
Schematics of the newly developed bias correction approach. Stars mark the grid cell under bias correction: dynamic LSM runoff time series are used for cumulative density function construction (blue line) and static runoff characteristics maps are used as the reference (red points). LSM = land surface model.

At each VIC grid cell (Figure 2), the model‐simulated daily runoff (35‐year, 12,784 samples) is used to construct the empirical CDF (blue line), with runoff values at different exceedance probabilities computed as *R*_99,m_, *R*_95,m_, *R*_90,m_, *R*_80,m_, *R*_50,m_, *R*_20,m_, *R*_10,m_, *R*_5,m_, and *R*_1,m_ (blue dots). The corresponding nine runoff characteristics, denoted as *R*_99,o_, *R*_95,o_, *R*_90,o_, *R*_80,o_, *R*_50,o_, *R*_20,o_, *R*_10,o_, *R*_5,o_, *R*_1,o_, respectively, are used as reference points for adjustment (red dots). To use the sparse reference information, the ratio correction factor *C*_*i*_ is calculated (equation (1)) at all available reference points. Assuming the intermediate ratio correction factors (*C*_*ij*_) between *C*_*i*_ and *C*_*i*+1_ follow a loglinear relationship (where *j* and *N* stand for the *j*th point and the total number of points between *i* and *i*+1, respectively; *i*+1stands for the next available runoff characteristics), *C*_*ij*_ can be written as equation (2). For modeled runoff values (*R*_m_) greater than *R*_1,*o*_ and those less than *R*_99,*o*_, a simple extrapolation technique is applied by taking the correction factor as *C*_99_ and *C*_1_. The bias‐corrected values are eventually computed by multiplying the original runoff time series by *C*_*ij*_. To avoid some rare cases of extremely large values (i.e., large model and observation mismatch seen in runoff values at 99% exceedance probability at ~4% of the cells), *C*_*ij*_ is capped at 1,000 after trial‐and‐error to ensure the corrected values are within a realistic range. The BC method is implemented using Python scripting; it is referred as “sparse CDF matching” and made available at https://github.com/peironglinlin/GlobalHydro/.

(1) $$C_{i}=R_{i,o}/R_{i,m}$$

(2) Cij=Ci1−j/N·Ci+1j/N,ifR99,o<Rm<R1,oC99,ifRm≤R99,oC1,ifRm≥R1,o

### 2.3 Vectorizing River Network and Unit Catchment from MERIT Hydro: MERIT Basins

Several preexisting global hydrography data sets (e.g., Lehner et al., 2006; Wu et al., 2012) can be used as the global routing data infrastructure. However, these data either lack coverages above 60°N (thus lacking arctic river basins), or their underlying DEM accuracy is limited in depicting realistic river planform geometry. In this study, we vectorize river flowlines and unit catchments from Multi‐Error‐Removed‐Improved‐Terrain (MERIT) Hydro, a recently developed high‐accuracy raster hydrography data (Yamazaki et al., 2019). The MERIT Hydro was based on MERIT DEM at 3‐arcsec (~90 m) resolution, which is an advanced high‐accuracy global DEM recently created by Yamazaki et al. (2017). MERIT Hydro precisely represents river networks as raster flow direction and flow accumulation from this DEM, providing a reliable hydrography at 3 arc‐second resolution with the full global coverage (60°S to 90°N). In order to tailor the MERIT Hydro for the modeling purpose that separates global basins, a few preprocessing steps are conducted. First, the Pfafstetter Level‐2 basin polygons from HydroBASINS (https://hydrosheds.org/images/inpages/HydroBASINS_TechDoc_v1c.pdf) are used to roughly divide the MERIT Hydro basins, because HydroBASINS jointly considers topographic controls and groundwater connections for its basin delineation (Lehner, 2014), providing a reasonable reference and coding protocol for hydrologic modeling. As MERIT Hydro provides data in 10°×10° tiles, for each basin, all intersecting MERIT tiles are first mosaiced as a single raster with sufficient buffer, where a 25 km^2^ channelization threshold is applied. Then, the vector flowlines and catchments are extracted from the 61 buffered mosaics by implementing TauDEM (http://hydrology.usu.edu/taudem/taudem5/index.html) with Princeton high‐performance computing to deal with the huge demand for computer memory. The resulting buffered data is then trimmed within Level‐2 basin boundaries by (1) locating the most downstream reaches within the HydroBASINS boundaries, (2) back‐tracing their contributing areas, and (3) redefining Level‐2 boundaries. Globally, there is less than 1% difference between MERIT Hydro and HydroBASINS Level‐2 basin boundaries on average; the final basin boundary is redefined based on MERIT Hydro. The vectorized hydrography (flowlines and unit catchments) within separated basin boundaries are referred as MERIT Basins v0.1 for future use. The 61 level‐2 MERIT Basins are further merged into nine Level‐1 continents (i.e., Africa, Europe, Siberia, Asia, Australia, South America, North America, Arctic of North America, and Greenland) for RAPID routing. See http://hydrology.princeton.edu/data/mpan/MERIT_Basins/ for more details and access of the MERIT Basins shapefiles derived in this study. It is worth noting that the median (mean) reach length is 6.8 km (9.2 km) and the unit catchment size is 36.8 km^2^ (45.6 km^2^). Although the routing algorithm adopted here (Muskingum) is of relatively low complexity, the underlying data infrastructure represents significantly improved accuracy and resolution for channel representation compared to existing global river modeling studies (e.g., Li et al., 2013; Renssen & Knoop, 2000; Sutanudjaja et al., 2018; Yamazaki et al., 2011). A visual comparison between the vector hydrography and the Google Map Images also suggest that the channel depictions are realistic enough to support locally relevant applications. In the future, the routing method can be updated with variable coefficient Muskingum‐Cunge (Lin, Hopper, et al., 2018) or other more advanced algorithms to further improve the surface water routing module.

### 2.4 Observational Data and Skill Metrics for Model Evaluation

**Figure 3:**
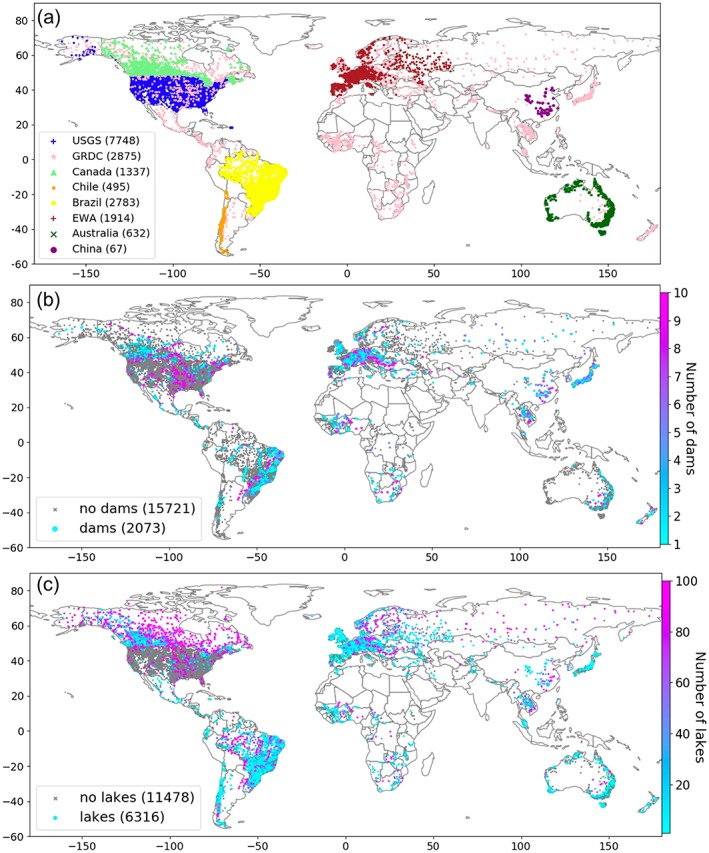
Global stream gauges used in model evaluation from (a) different gauge networks. Gauges are classified based on if they are influenced by (b) dams and (c) lakes or not. If influenced by dams/lakes, colors show the number of dams/lakes upstream. USGS = U.S. Geological Survey; GRDC = Global Runoff Data Centre.

To evaluate the simulated discharge, we use daily streamflow observations obtained from a global database we compiled from multiple national and international sources (see Acknowledgements for more details), which covers >17,000 gauges globally (Figure 3). Not surprisingly, these data are unevenly distributed with richer data in developed countries than other parts of the world (Figure 3a). Due to inconsistent data collection and sharing policies, these data also differ significantly in total record length and data quality (e.g., the U.S. Geological Survey uses “quality tags” to inform measurements quality while others do not and may contain poor data). Nevertheless, to make the most use of the data to reveal the model performance to the maximum spatial extent possible, over 14,000 gauges with more than 3 years of data (not necessarily consecutive) are used for model validation. The gauges are snapped onto the MERIT Basins flowlines with a “spatial join” analysis—gauges located >500 m from its closest reach are considered to have poor coordinate information and are eliminated from evaluation (lowering the snapping threshold may more accurately locate gauges for small reaches, but we found very few wrong locations when comparing the gauges' documented drainage areas with the reaches' calculated areas, so it is adopted to retain more gauges in a consistent manner).

To further classify the gauges into those influenced by lakes or dam regulations and those located in naturalized basins with no lake or dam impacts, the Global Reservoir and Dam point data set (GRanD v1.01, http://sedac.ciesin.columbia.edu/data/set/grand-v1-dams-rev01) and the HydroLAKES polygon data set (Messager et al., 2016) are used as the ancillary data. GRanD compiles 6,862 dams globally and HydroLAKES compiles 1.4 million natural lakes. We delineate the upstream drainage basins of all gauges and intersect them with the ancillary data–this determines how many GRanD dams (Figure 3b) and HydroLAKES natural lakes (Figure 3c) are located in the drainage basin of each gauge. The gauges are thus separated into three groups: (1) naturalized gauges with no influences from lakes/dams (11,313), (2) gauges influenced by reservoir regulations (2,073), and (3) gauges with influence from lakes (6,316). Geographically, gauges with large numbers of upstream dams are mostly located in large rivers with higher Strahler stream orders, corresponding to the places that have the most human impacts (Figure 3b). Gauges with large numbers of upstream natural lakes (Figure 3c) are located in the tropics (due to large precipitation inputs) as well as the high‐latitude river basins (due to lake formation during the glacial‐interglacial geologic cycles).

**Figure 4:**
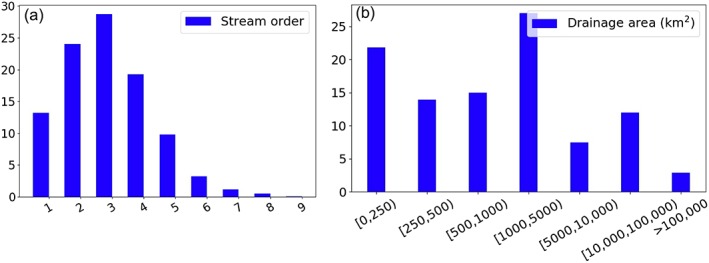
Histogram of (a) stream order and (b) drainage basin area (km^2^) of the gauges shown in Figure 3a.

These gauges cover the full spectrum of stream orders (Figure 4a), and they also range greatly in drainage basin size (see Figure 4b)—the median and mean of the drainage area sizes are 965.1 km^2^ and 18,613.9 km^2^, respectively. Since this study uses a highly accurate hydrography derived from the 90‐m DEM for river routing, we are able to include gauges located in headwater streams with very small drainage basins (e.g., 22% of the gauges have a drainage area of <250 km^2^) in the model evaluation. This is in contrast to previous global studies that mainly performed their evaluation for large drainage basins (Decharme et al., 2010; Li et al., 2013). The inclusion of all gauges in the evaluation poses more challenges toward the “hyperresolution” goal of any global river modeling framework, and thus both excellent and poor simulations are expected at the scale the model is assessed.

To comprehensively evaluate the model, the Kling‐Gupta Efficiency (KGE, equation (3)) and its component statistics including correlation coefficient (CC, equation (4)), bias ratio (BR, equation (5)), and relative variability (RV, equation (6)) are calculated at both daily and monthly time scales. In the equations, *Q*_*m*_ and *Q*_*o*_ denote modeled and observed discharge, respectively; $\sigma_{Q_{m}}$ and $\sigma_{Q_{o}}$ denote standard deviation of the modeled and observed discharge, respectively. KGE measures the Euclidean distance between a point and the optimal point that has CC = 1, BR = 1, and RV = 1 (Gupta et al., 2009; Kling et al., 2012). Therefore, it is an integrated skill metric to jointly consider the modeled time series covariability with the observation, the model bias, and the model standard error, and is widely used in evaluating environmental models (Gupta et al., 2009). Any approach that attempts to maximize the KGE is a multiobjective calibration process that simultaneously optimizes CC, BR, and RV. In the following sections, we purposefully do not differentiate gauges influenced by water management and those gauges located in naturalized basins in the spatial plot analyses (sections 3.1–3.3); a closer look at the influence from reservoirs/lakes, drainage basin area, and climatic aridity index will be separately conducted through boxplot analyses in section 3.4.

(3) $$\mathrm{KGE}=1-\sqrt{{\left(\mathrm{CC}-1\right)}^{2}+{\left(\mathrm{BR}-1\right)}^{2}+{\left(\mathrm{RV}-1\right)}^{2}}\;$$

(4) $$\mathrm{CC}=\frac{\mathrm{cov}\left(Q_{m},Q_{o}\right)}{\sigma_{Q_{m}}\cdot \sigma_{Q_{o}}}$$

(5) $$\mathrm{BR}=\bar{Q_{m}}/\bar{Q_{o}}$$

(6) $$\mathrm{RV}=\left(\sigma_{Q_{m}}/\bar{Q_{m}}\right)/\left(\sigma_{Q_{o}}/\bar{Q_{o}}\right)$$

## 3 Results

### 3.1 Baseline Model Simulation Performance

**Figure 5:**
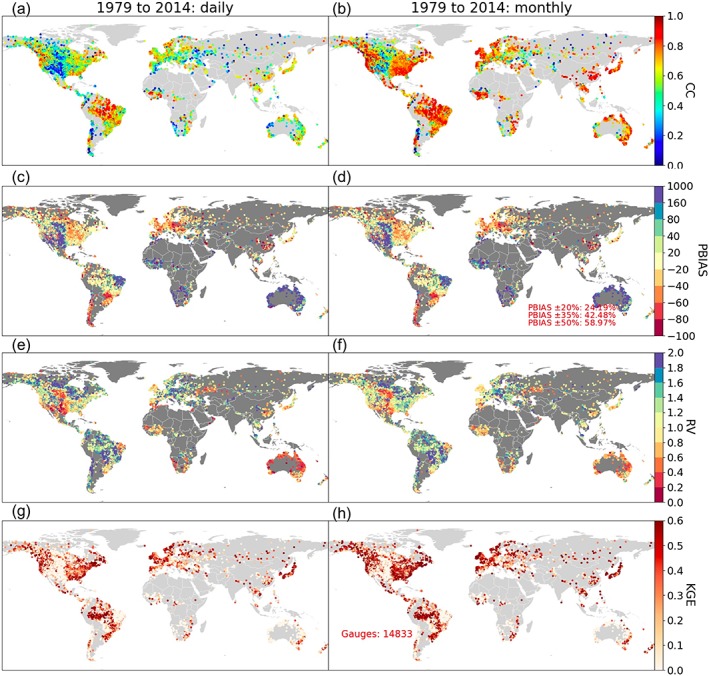
Global streamflow modeling evaluated against over 14,000 gauges (left column: daily; right column: monthly). Results shown are correlation coefficient (a, b), percentage bias (c, d), relative variability (e, f), and the Kling‐Gupta Efficiency (g, h). Note the CC plot has a range of 0 to 1 because only ~1% of gauges have negative CC; the PBIAS plot has asymmetric ranges because of its definition that can result into asymmetric values for dry and wet biases. CC = correlation coefficient; RV = relative variability; KGE = Kling‐Gupta Efficiency; PBIAS = percentage bias.

Figure 5 shows the geographic distribution of the CC, percentage bias (PBIAS = ( $\bar{Q_{m}}-\bar{Q_{o}})/\bar{Q_{o}}\times 100\%$), RV, and KGE skill metrics. Overall, the model shows better performance in humid regions than dry regions. The monthly statistics (right column) are better than the daily KGE scores (left column)—the difference is mainly a result of the CC difference, suggesting daily flow dynamics are less well captured than monthly flows. This is partly related to the difficulty for the forcing to capture the dynamic daily precipitation variability, and partly due to the use of the simple Muskingum routing method that cannot fully resolve daily flow variations. Solving the full Saint‐Venant equations may improve the simulation of the daily flow fluctuations, but the performance will still depend on the channel geometry parameterizations required by the hydrodynamic equations. For the purpose of providing a priori information for SWOT, however, we consider the relatively simple routing scheme sufficient because the monthly and climatological flow is of the primary focus in this study. Over majority global land areas, CC is in the range of 0.6 to 1 (monthly) and 0.4 to 0.8 (daily) except for a few complex‐terrain areas such as Chile, the Alps, and the central to western United States. Such a result is generally better than those when evaluating ten state‐of‐the‐art hydrologic models for discharge modeling (Beck et al., 2017). Compared to CC, the model bias problem is more pronounced, and only 24.2% of the gauges have a PBIAS within ±20%—not knowing the observational data quality (Kiang et al., 2018), a PBIAS within ±20% is used to delineate geographic locations showing very good model performances. For the mostly dry central United States, Africa, and Australia, the model exhibits >100% PBIAS (blue color); this problem is general in a couple of other LSMs as well (e.g., Xia et al., 2012). For the western United States, Europe, and eastern Asia, the model slightly underpredicts runoff (see red colors for the negative model biases). The biases cause unsatisfactory KGEs in both daily and monthly simulations (i.e., white dots with negative KGEs; Figures 5g and 5h), and they need to be resolved before the database can be used in serving other hydrologic applications, as performed here and discussed next.

### 3.2 Grid‐by‐Grid BC Against Runoff Characteristics

**Figure 6:**
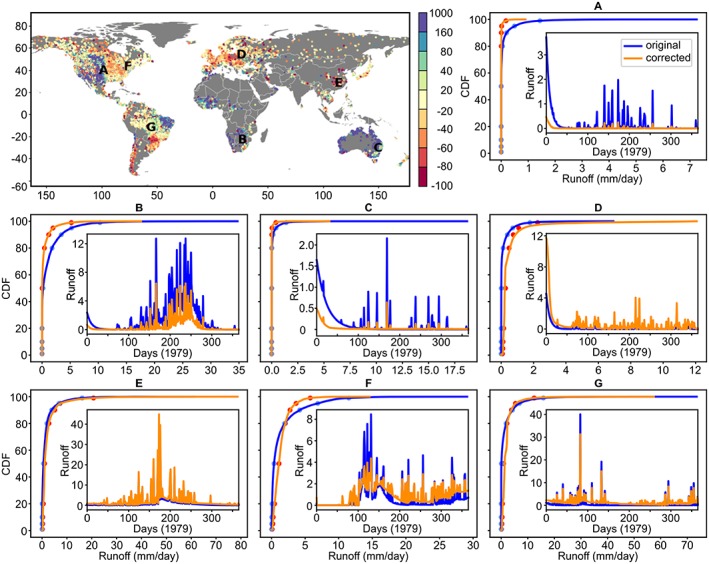
Effectiveness of the bias correction approach against nine runoff characteristics. The spatial plot shows the percentage bias (%) of the original model simulation; (a)–(g) show the CDF and time series change before (blue) and after bias correction (orange) at seven example grid cells. For clarity, only the runoff time series for Year 1979 are shown. CDF = cumulative density function.

To deal with the model biases, the sparse CDF matching introduced in section 2.2 is implemented at all ~0.24 million VIC grid cells. Figure 6 illustrates the BC effectiveness by showing the runoff time series and the CDF changes before (blue) and after the correction (orange) at seven example grid cells. These cells exhibit different types of bias problems. Regions with over‐predicted runoff problems, such as central United States (A), Africa (B), and Australia (C), have seen lowered runoff after BC (Figures 6a–6c). BC reduces the positive biases because the runoff signatures above *Q*_50_ estimated by ML are much lower than the model simulation (Figures 6a–6c). Over regions with underpredicted runoff such as Europe (D) and eastern Asia (E, red dots in the original bias map), runoff time series are corrected to match the systematically higher *Q*_c_ estimates (Figures 6d and 6e). For regions where the model originally shows good model performances such as the eastern United States (F) and the Amazon River basin (G, see yellow dots in Figures 6f and 6g), the influence of BC is minimal particularly on the mean/median flows (i.e., very little change in flows around *Q*_50_ before and after BC). This suggests the VIC LSM and the ML‐derived *Q*_c_ maps provide similar mean/median runoff estimates over these areas, which implies it is generally less challenging to understand the hydrology there. Note that the BC approach is not only effective in addressing systematic model biases such as Point A through Point E but also able to adjust different runoff percentiles by lowering high flows and increasing low flows (e.g., Point F and Point G, where the fourth and third last reference points, respectively, are break points to deviate from model‐simulated low flows and high flows). The log‐linear assumption (equation (2)) avoids the sudden change in the ratio correction factors, which leads to the overall smooth corrections of the CDF in matching the ML‐derived *Q*_c_ maps. A global difference map before and after BC is shown in Figure S4.

### 3.3 Global Streamflow Simulation After BC

**Figure 7:**
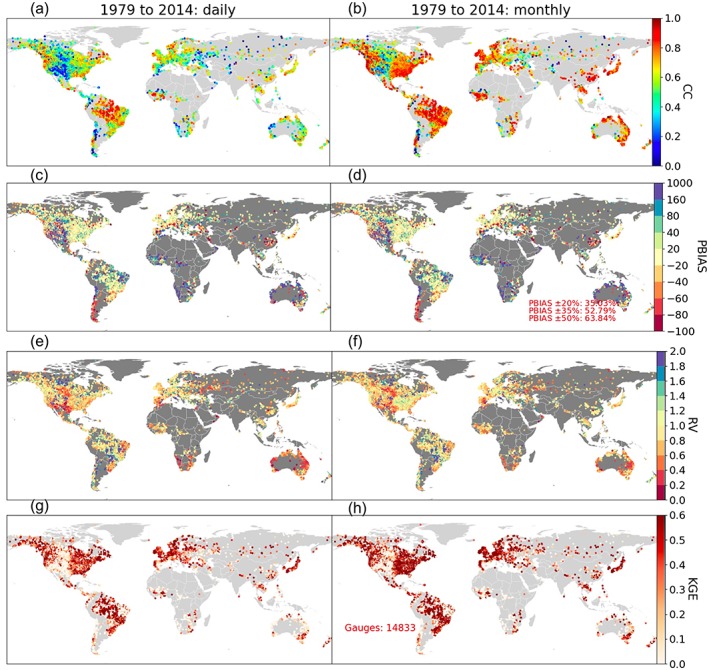
The same as Figure 5, but for the simulation performance after bias correction.

To further evaluate the BC effectiveness, the corrected runoff is routed with RAPID and the resultant model performance is shown in Figure 7. Among all gauges, now 35% have a PBIAS within ±20% (yellow dots in Figures 7c and 7d), and 29% (62%) have a monthly KGE greater than 0.6 (0.2). The best KGEs are seen over the eastern United States, South America, Europe, and eastern Asia, all generally humid regions, while the model still struggles to produce satisfactory KGEs for central United States, eastern South America, parts of Africa, and Australia (see white dots in KGE plot). The negative KGEs are partly related to the runoff biases still present after BC (Figures 7c and 7d), and partly related to the consistently low CC (Figures 7a and 7b) that cannot be adjusted unless better flow dynamics are simulated using better precipitation forcing or updated routing methods. It is noteworthy that these spatial plots do not differentiate gauges influenced by lakes/reservoirs and those with small drainage basins (or very small, narrow rivers). Thus, gauges whose mean flow and flow dynamics are by nature difficult to simulate in a perfect sense have not been screened out, and the aim is to reveal, to the maximum extent possible, the capabilities and limitations with the presented model. These issues are not expected to be resolved by the proposed BC approach and will need to be addressed using improved‐accuracy precipitation inputs, and/or well‐represented and calibrated water management modules, which is not yet available to the framework. These are also the places where RS observations like those from SWOT may make a contribution by providing new primary data and independent estimates of the river flow. Problems associated with the *Q*_c_ maps (Beck et al., 2015) may also impede a perfect skill improvement everywhere, for example, the bias can be increased at certain gauges (see next section). Future studies are warranted to refine the data/methods/models by addressing the above‐mentioned issues. Nevertheless, given the availability of high‐quality global data sets and limited information in ungauged basins, the improvements across a large spatial scale after the proposed BC still demonstrate promising results, upon which future refinements could be conducted.

**Figure 8:**
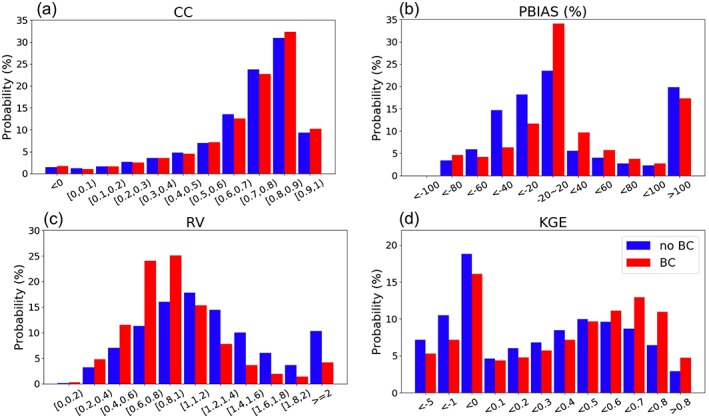
Histogram of the (a) CC, (b) PBIAS, (c) RV, and (d) KGE skill changes before (blue) and after BC (red bars) for the monthly simulation. CC = correlation coefficient; RV = relative variability; KGE = Kling‐Gupta Efficiency; PBIAS = percentage bias; BC = bias correction.

The bulk statistics for the 14,000 gauges (Figure 8) show that BC is able to increase the number of gauges that have a PBIAS within ±20% by 11% (Figure 8b), while shifting the peak KGE to 0.6–0.7 (red bars) from 0.4–0.5 previously (blue bars) in Figure 8d. CC is barely changed (Figure 8a) and gauges with RV between 0.8 and 1.2 are also increased (Figure 8c). Despite the promising improvements, there are still gauges consistently showing negative KGEs (although less after BC). These are mostly for extremely dry rivers (see section 3.4), where a slight overestimation by the model (e.g., 0.1 mm) would easily produce a large percentage error. Hence, although BC is able to reduce the absolute amount of the overestimated runoff amount (e.g., Figures 6a–6c), the remaining bias still takes up a large percentage, resulting into negative KGEs. There is also a tendency for BC to shift the PBIAS to the higher end (Figure 8b), which implies that the ML‐derived *Q*_c_ maps provide consistently higher mean runoff estimates than the model (although not at the highest end), which is a place needing refinements in the future. The fact that the model is not representing transmission loss in dry regions can also partly explain the stubborn bias. In addition, water management activities such as diversions and irrigations are neither parameterized by the model nor implicitly incorporated in the *Q*_c_ maps (only naturalized flows were used for ML training; see Beck et al., 2015). Therefore, room is left for further improvements in future studies.

### 3.4 Dam Regulation, Lake Impacts, Drainage Basin Area, and Aridity Index

To better understand the spatially explicit model performance, this section presents more analyses that differentiate the evaluation gauges based on whether or not they have dam/lake impacts, their drainage basin size, and their climate aridity.

**Figure 9:**
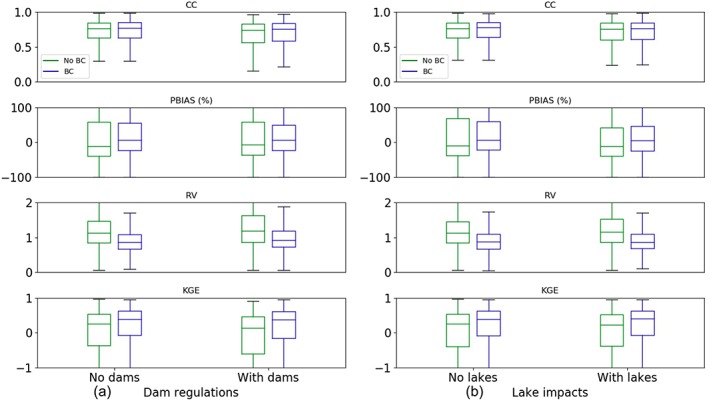
Boxplot of the model performance statistics based on (a) dam regulation and (b) lake impacts. The left (right) column in each plot shows gauges with (without) the influence from dams or lakes. Statistics for experiment with no BC and that with BC are shown in green and blue boxes, respectively. CC = correlation coefficient; RV = relative variability; KGE = Kling‐Gupta Efficiency; PBIAS = percentage bias; BC = bias correction.

In the “no BC” experiment, whether or not a gauge has upstream natural lakes only minimally influences the model performance (i.e., no significant difference between left and right groups of Figure 9b). The existence of dams (Figure 9a), on the other hand, is slightly more influential as can be seen from the higher median PBIAS and RV in the “With dams” group and the slightly higher (narrower) KGEs in the “No dams” group (green boxes in Figure 9a). This suggests that dams are more effective in altering flow dynamics than natural lakes, which is because natural lakes (especially small ones) can behave like wide river channels while dams are usually regulated by humans to counteract natural flow variability. Consequently, the current routing model might benefit more from incorporating a well‐represented and calibrated global dams/reservoirs scheme compared to adding a scheme for lake processes. After BC, we see the model performance differences between gauges with and without the influence from lakes or dams both become less prominent (blue boxes in Figure 9) with improved model performance. This indicates that BC is able to reduce some biases resulted from the underrepresented lakes/reservoirs in the routing model. Overall, the difference between gauges with and without upstream lakes/reservoirs is surprisingly less prominent than expected. This may be partly related to the fine scale at which the model is assessed, which makes other existing problems more outstanding than the lack of reservoir or lake schemes (as will be discussed below). It may also be partly ascribed to the uncertainty in the dams/lakes datasets used for the analyses, for example, GRanD only accounts for large dams, but recent studies suggested that those small yet much more widespread reservoirs, especially small farm dams to draw water from rivers for irrigation, can have a strong cumulative impact on hydrology (Habets et al., 2018). In addition, our analyses did not account for distance between the dams/lakes and the gauges. These issues will need to be more carefully investigated in the future with improved data sets.

**Figure 10:**
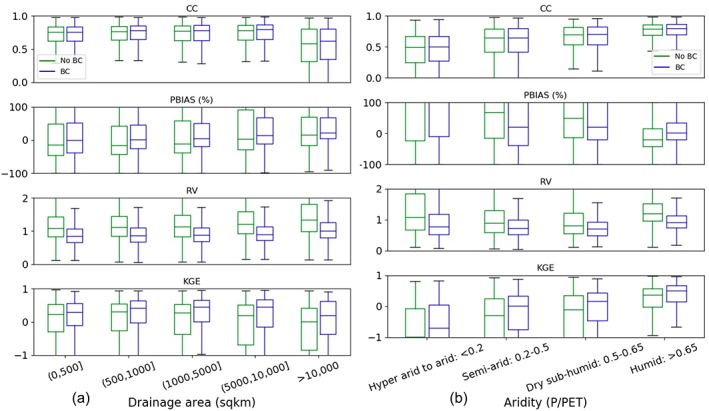
Same as Figure 9, but for (a, left) drainage area and (b, right) aridity.

We next show the model performance as a function of the drainage basin size and the climate aridity (Figure 10). In the BC experiment, the median KGE slightly increases with drainage size for basins smaller than 5,000–10,000 km^2^, which is because the model errors of various sources tend to be canceled out as the drainage basins aggregate in size (Cunha et al., 2012; Lin, Yang, et al., 2018). The median KGE becomes slightly lower for basins greater than 10,000 km^2^, which is most likely related to the dam existence that makes the model to produce high‐biased flows (Figure 10a, PBIAS) and flow variabilities (lower CC, higher RV in Figure 10a); note that the lower KGEs are more obvious for no BC experiment for drainage areas greater than 5,000 km^2^. Among all factors analyzed (i.e., lakes, dams, drainage area, and aridity), the most pronounced challenge for the model simulation lies in the dry region runoff prediction (Figure 10b), where both the flow dynamics (median CC < 0.5, median RV > 1.5) and the mean flow (median PBIAS > 100%) are difficult to resolve compared to humid regions (median CC > 0.6, median PBIAS < 20%, median KGE > 0.5). This problem, although alleviated after BC (Figure 10b), is still the most outstanding issue. These results can be used to prioritize, among many potential pathways for model improvements, the most important tasks as the immediate next step. Here we suggest to largely improve the geographically distributed model performance, future studies tailored to dry regions are critical. For the purpose of providing a priori river flow information for SWOT, however, this may be less of an issue because most dry region rivers will be not observable from SWOT (section 3.5).

### 3.5 Potential Applications to the SWOT Mission

**Figure 11:**
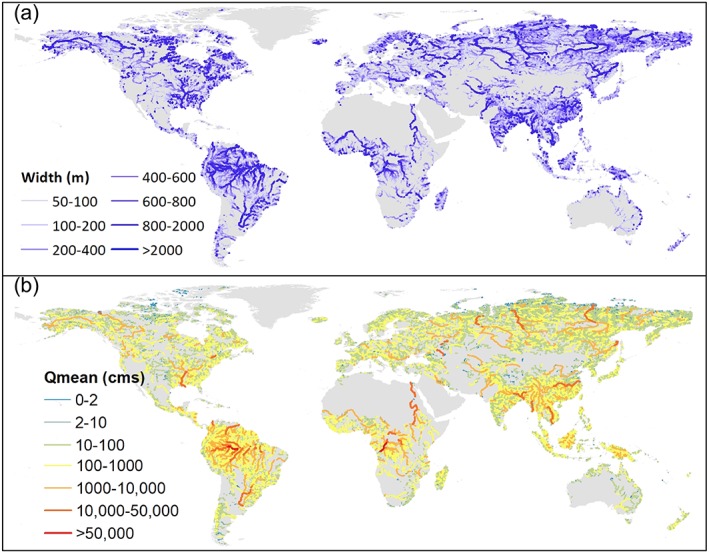
(a) The global river width and (b) the Variable Infiltration Capacity‐Routing Application for Parallel computatIon of Discharge‐simulated mean annual discharge for the river reaches observable by SWOT (width >50 m, as determined using GRWL)—there are 211,968 such reaches, taking up 8.8% of the total modeled reaches. Cross‐sectional width measurements are obtained from the GRWL database (Allen & Pavelsky, 2018) and are averaged over the Multi‐Error‐Removed‐Improved‐Terrain reaches, excluding measurements with lake/reservoir tags and multichannels. Note that SWOT will not observe a handful of reaches north of 78°N, but they are not differentiated here. GRWL = Global River Widths from Landsat; SWOT = Surface Water and Ocean Topography.

As mentioned in section 1, SWOT discharge estimates will be computed using simple flow laws such as Manning's equation where some terms in the equation are directly observable, and some parameters (e.g., river bathymetry and friction parameter) are not observable. Thus, the inversions are often ill posed and will rely upon an a priori estimate of river flows. Our presented 35‐year model‐estimated river discharge would provide one such estimate of a priori river flow, to help constrain the inversion algorithms. In this section, we cast our results in terms of SWOT‐observable reaches to demonstrate the potential application of the final data record. Note that SWOT will only observe rivers wider than 100 m, with a target of 50 m (Pavelsky et al., 2014), which is a subset of the spatiotemporally continuous global discharge record presented in this study. We use a new observation‐based global river width data set—the Global River Widths from Landsat (GRWL) database (Allen & Pavelsky, 2018) to determine which modeled reaches can be seen by SWOT. GRWL used over 7,000 Landsat scenes to calculate channel widths for reaches wider than 30 m (i.e., the Landsat pixel resolution) at their mean annual discharge. It provides >58 million width measurements orthogonal to the Landsat centerlines. To map these “cross‐sectional” widths onto the MERIT Basins reaches (median length 6.8 km), geospatial analyses are performed by (1) spatially joining GRWL point measurements with the flowlines (excluding measurements located >100 m away from its closest flowline), (2) excluding GRWL measurements tagged with lakes, reservoirs, and multiple channels, and (3) calculating the reach‐averaged widths only if a MERIT reach has more than 10 GRWL measurements to reduce possible noisy data. The MERIT Basins flowlines are then enhanced with width measurements informed from GRWL. The spatial patterns of the global reach‐based “effective” river widths and the mean annual discharge computed from the best modeling experiments from this study are shown in Figure 11.

For all river reaches potentially observable from SWOT taking up 8.8% of our modeling database (Figure 11a), the newly reconstructed flow record shows even better statistics as evaluated against ~4,000 gauges: 44% (76%) of them have a PBIAS within ±20% (±50%), and 42% (75%) have a monthly KGE greater than 0.6 (0.2). This gives us confidence in using the model‐derived flow characteristics such as mean annual discharge (Figure 11b) in a range of established SWOT discharge algorithms (e.g., those in Durand et al., 2016; Hagemann et al., 2017). Since this modeling database involves the flow duration curve across the 35‐year time span, it can also provide flow uncertainty needed by some algorithms, or it can be extended to ingest multi‐LSMs, multiple forcings, and parameter perturbations in the future for more rigorous flow uncertainty estimation. Daily, monthly, and seasonally varying flow estimates may also be used to improve the SWOT discharge accuracy, beyond the static *Q*_MEAN_ currently used (Durand et al., 2016)—Figure S5 shows the model performance statistics as separated by season. Newly developed discharge estimation methods such as Bjerklie et al. (2018) rely on flow quantiles, e.g., discharge with a 2‐year recurrence interval as an approximation for bankfull discharge, which can also be directly calculated from our presented database. Overall, the updated framework/methodology shows robustness in estimating global river flows “everywhere and locally relevant,” synthesizing the most recent advancements in precipitation merging, LSM calibration, LSM BC, and hydrography developments (section 2.1). Moreover, it addresses the fine‐scale channel depiction needs as required by the SWOT satellite mission and biogeochemical applications (Downing, 2012). Together with the spatially‐explicit accuracy assessment as shown in Figure 7, we expect the database to be used by RS and modeling groups in an iterative manner in the future, that is, hydrologic modeling provides updated initial estimates to improve RS estimates and RS provides constraints to update the modeling parameters and simulations through data assimilation, to jointly improve our quantification of the global river flow dynamics.

Lastly, it is also interesting to note the reach‐averaged width pattern in Figure 11a shows some deviations from another reach‐based global river width database derived from the traditional downstream‐hydraulic‐geometry (DHG) approach (Andreadis et al., 2013). This implies that the simplified DHG theory may be inadequate at the scale of the state‐of‐the‐art RS and modeling (i.e., 30 to 90 m), as was also discussed by Allen and Pavelsky (2018). Using the updated global discharge database and the newly available global river width data set, the DHG theory can be revisited, similar to a recent study by Frasson et al. (2019), but with improved water balance accounting and covering rivers beyond 60°N. Updating the theoretical understanding of DHG, or adopting different geomorphological paradigms, may potentially further our understanding of the spatial variability of global river widths.

## 4 Conclusions and Discussions

This study presents a carefully designed modeling effort for global river discharge simulation at unprecedented 2.94 million reaches. To constrain the simulation as much as possible, we (1) utilize a newly developed 0.1° global precipitation dataset that optimally merged a range of global gauge‐, satellite‐, reanalysis‐based data sets, (2) conduct grid‐by‐grid (instead of basin‐by‐basin) VIC model parameter calibration, (3) bias‐correct the runoff simulation with a newly proposed “sparse CDF matching” approach, and (4) extract vector river flowlines from the high‐accuracy 90‐m global MERIT DEM covering 60°N and above (our resulting vector hydrography is named “MERIT Basins”) for river routing. The 35‐year daily and monthly model simulation (1979 to 2014) are comprehensively evaluated against >14,000 gauges globally covering the full spectrum of stream orders, results of which demonstrates the robustness and high‐accuracy of the data record for use in SWOT and other hydrologic applications—the new flow record is named “Global Reach‐Level A Priori Discharge Estimates for SWOT (GRADES).” “MERIT Basins” and “GRADES” will be made publicly available for research purposes at http://hydrology.princeton.edu/data/mpan/MERIT_Basins/ and http://hydrology.princeton.edu/data/mpan/GRADES/.

Despite the promise of the newly reconstructed global river flow records, we believe there are still several areas where future refinements can be conducted. First, as presented in the Results section, the modeling performance can still benefit from improving the dry region simulations, adding reservoir regulation modules, updating the routing method with advanced algorithms, and adding modules for lake processes (the order of appearance reflects the modeling priority in future work). Second, although our goal is to estimate river flows across the full spectrum of stream orders and this goal is better approached compared to previous global modeling studies, many headwater streams important for freshwater biogeochemical cycling (Benstead & Leigh, 2012) may still not be explicitly modeled. Flow estimation for those headwater streams will need to be made possible with future advancements in global LSM and DEM at higher spatial resolutions, combined with insights gained via new in situ observations (Allen, Pavelsky, et al., 2018). Third, the *Q*_c_ maps used for BC and model calibration contain some “observational information” from a small subset of the final evaluation gauges, and they are assumed as the reference despite these maps are not free from errors (Beck et al., 2015). Future work may revise this assumption in order to achieve additional skill increment on top of the BC method presented. Finally, local errors may still be present, but we do not aim to address these issues unless other high‐quality global datasets become available to serve as the reference. Future improvements could benefit from in‐depth analyses of the uncertainty with runoff characteristics maps (Beck et al., 2013, 2015), the model performance in arid regions (Newman et al., 2015; Parajka et al., 2013; Pilgrim et al., 1988; Ye et al., 1997), and the precipitation forcing uncertainty (Alijanian et al., 2017; Beck et al., 2017, 2019; Satgé et al., 2019). Based upon the reconstructed naturalized river flows presented in this study, we expect future iterative efforts between modeling and RS groups, that is, updated models provide better a priori flow information to the RS and RS provides constraints to the model parameters and simulations through data assimilation, to jointly improve our quantification of the global river flows.

## Supporting information



Supporting Information S1
