# Supplementary material for: Global Reconstruction of Naturalized River Flows at 2.94 Million Reaches
Source: Water Resour Res. 2019 Aug 5;55(8):6499–516. doi: 10.1029/2019WR025287 (PMC6853258; doi:10.1029/2019WR025287)
Supplement: Supplementary file 1 — Supporting Information S1 [file WRCR-55-6499-s001.docx]

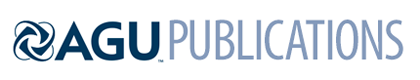


*Water Resources Research*

Supporting Information for

**Global reconstruction of naturalized river flows at 2.94 million reaches**

Peirong Lin^1,*^, Ming Pan^1,*^, Hylke E. Beck^1^, Yuan Yang^1,2^, Dai Yamazaki^3^, Renato Frasson^4^, Cédric H. David^5^, Michael Durand^4^, Tamlin M. Pavelsky^6^, George H. Allen^7^, Colin J. Gleason^8^, Eric F. Wood^1^

1. Department of Civil and Environmental Engineering, Princeton University, Princeton, New Jersey, USA

2. State Key Laboratory of Hydroscience and Engineering, Department of Hydraulic Engineering, Tsinghua University, Beijing, China

3. Institute of Industrial Science, The University of Tokyo, Tokyo, Japan

4. School of Earth Sciences, The Ohio State University, Columbus, Ohio, USA

5. Jet Propulsion Laboratory, California Institute of Technology, Pasadena, California, USA.

6. Department of Geological Sciences, University of North Carolina Chapel Hill, North Carolina, USA

7. Department of Geography, Texas A&M University, College Station, Texas, USA

8. Department of Civil and Environmental Engineering, University of Massachusetts, Amherst, Massachusetts, USA

**Contents of this file**

Text S1

Figures S1 to S3

**Text S1.** The three calibrated VIC parameters are the variable infiltration curve parameter (b), thickness of soil layer 2 (thick2), and fraction of the maximum velocity of base flow at which nonlinear base flow begins (Ds). b controls the shape of the variable infiltration curve in VIC, and effectively dictates the partitioning of rainfall into infiltration and surface runoff. Thick2 determines the size of the infiltration reservoir. Ds is a parameter that affects the base flow by controlling when nonlinear base flow will happen. Runoff generation in VIC is highly sensitive to the three parameters (Demaria et al., 2007).

**
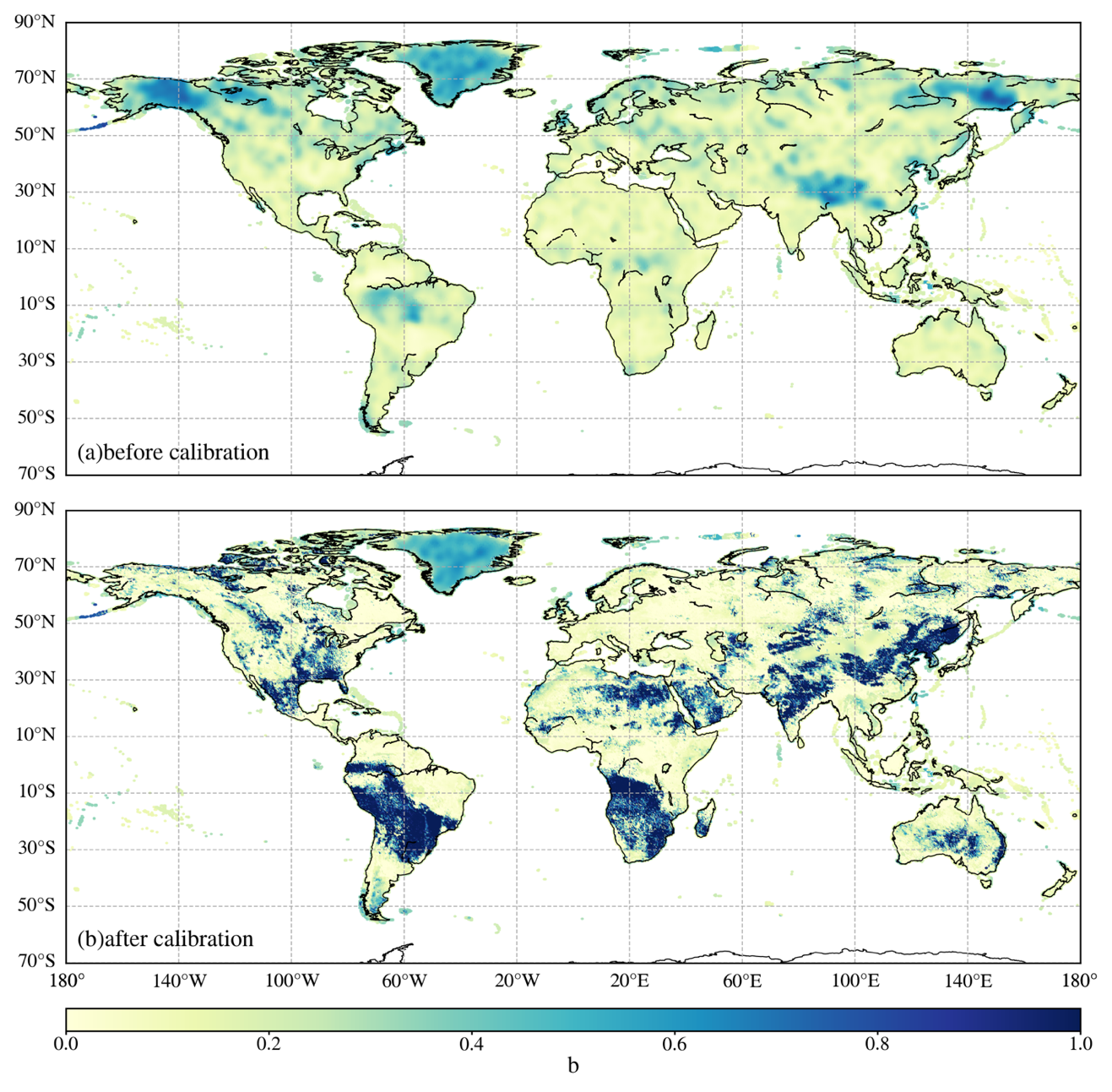
**

**Figure S1.** Spatial distribution of the *b* parameter of the VIC LSM at the global scale (0.25$^{\circ}$): (a) before and (b) after calibration.

**
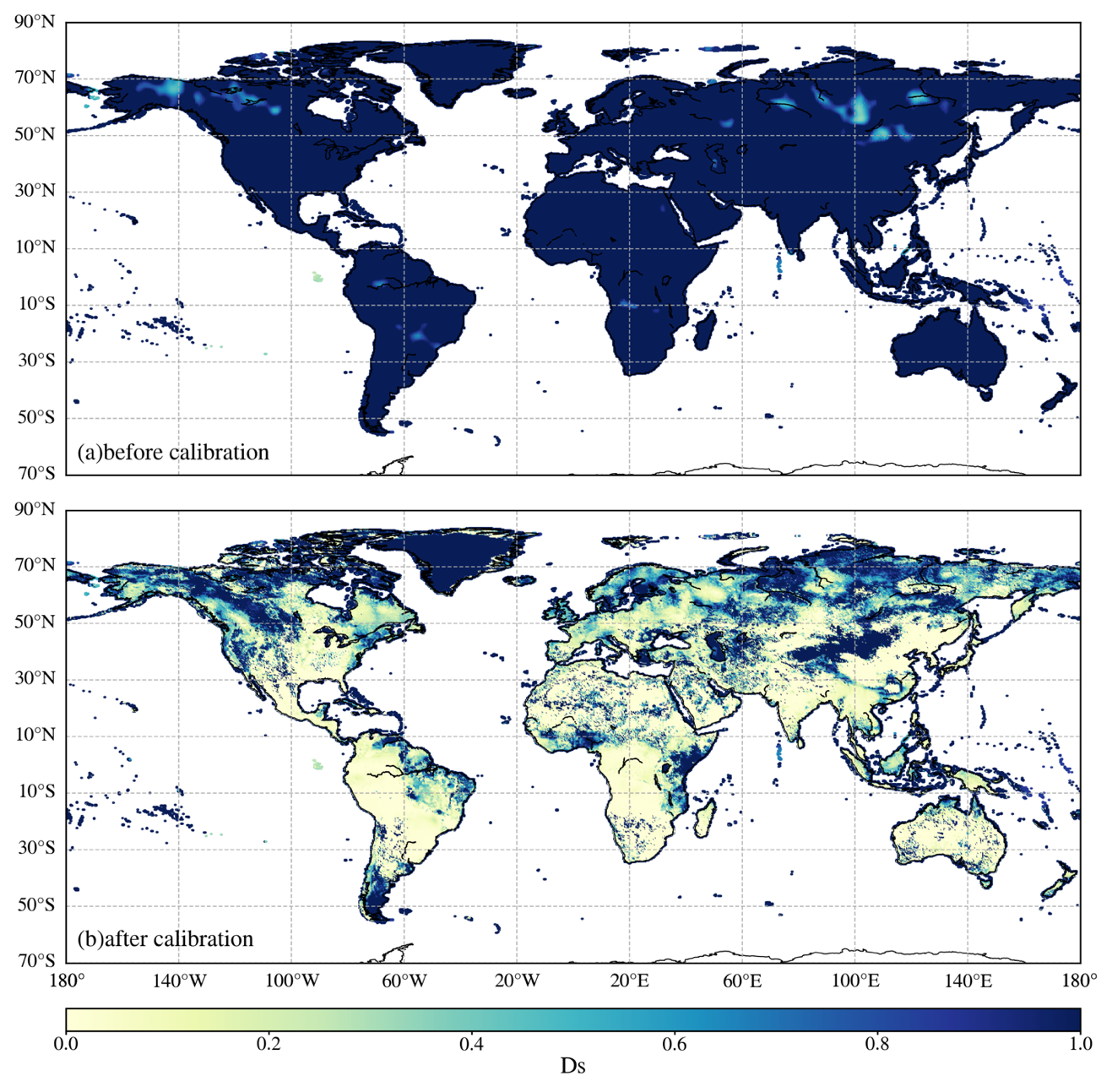
**

**Figure S2.** The same as Fig. S1, but for *Ds*.

**
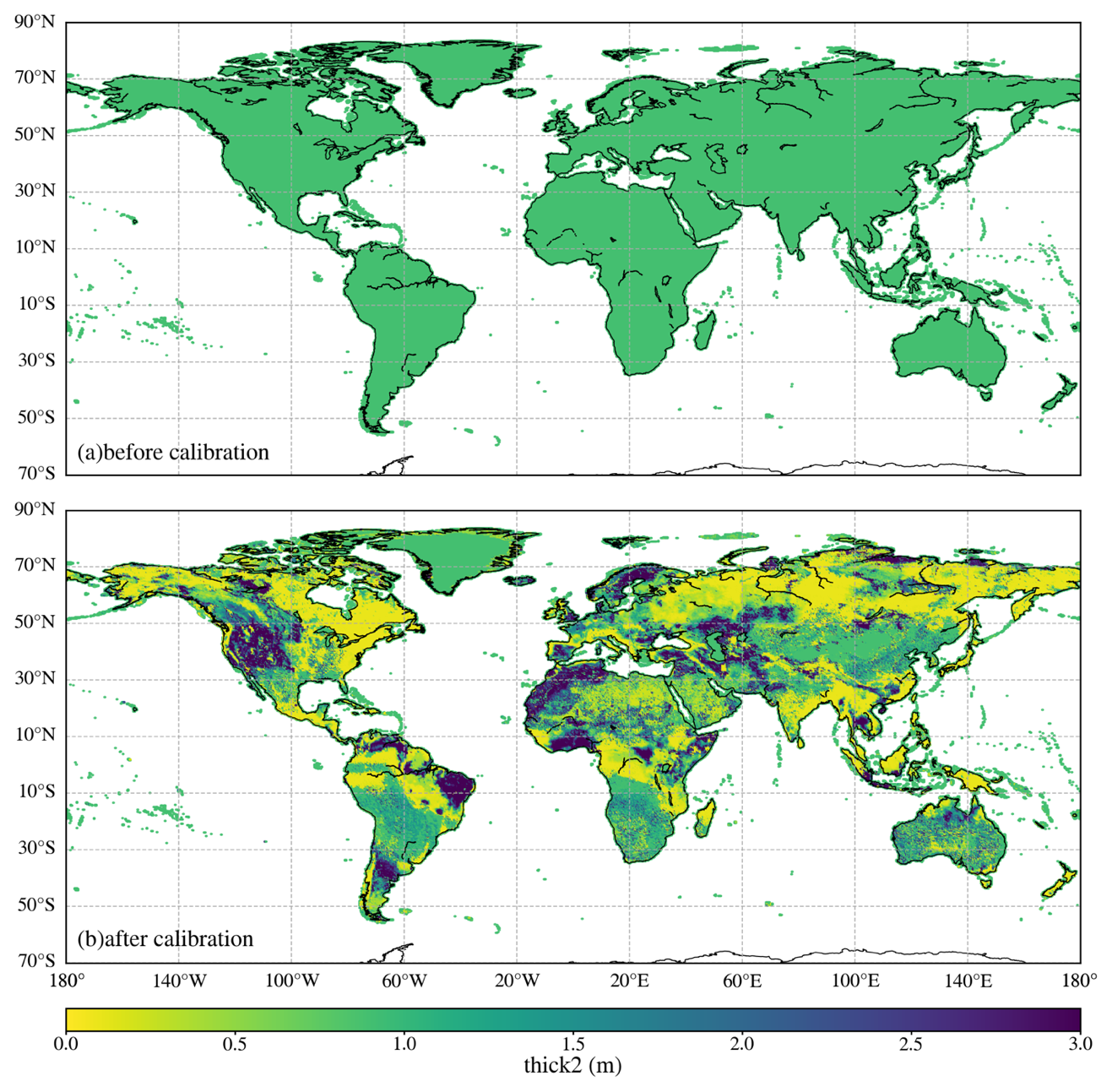
**

**Figure S3.** The same as Fig. S1, but for *thick2*.


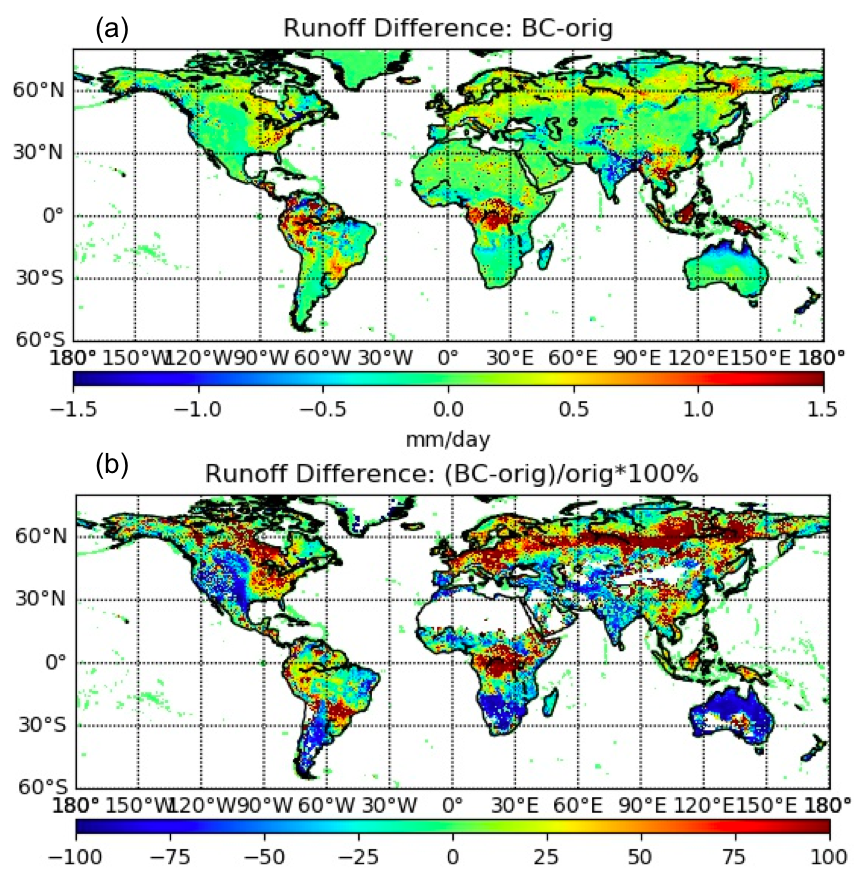


**Figure S4.** Runoff difference between BC vs. no BC experiment: (a) absolute difference (mm/day) and (b) percentage difference (%). Regions with runoff less than 0.05 mm/day are masked. 39% of the land grid cells show less than $\pm$20% change in runoff after BC.


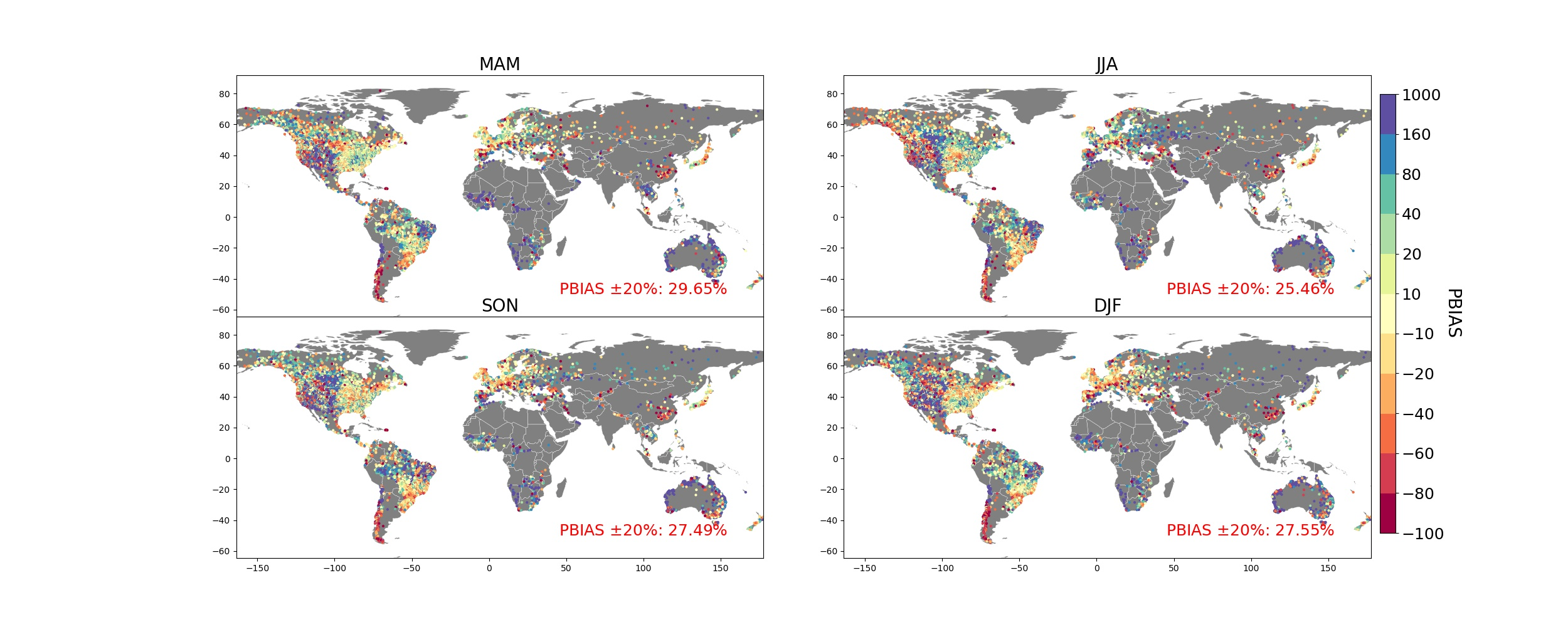


**Figure S5.** Model performance statistics as separated by season (MAM: March-April-May, JJA: June-July-August, SON: September-October-November, DJF: December-January-February). Yellow colors show gauges where PBIAS between model and observation is less than $\pm10$%.
